# Damage Detection of L-Shaped Concrete Filled Steel Tube (L-CFST) Columns under Cyclic Loading Using Embedded Piezoceramic Transducers

**DOI:** 10.3390/s18072171

**Published:** 2018-07-06

**Authors:** Juan Zhang, Yong Li, Guofeng Du, Gangbing Song

**Affiliations:** 1 School of Urban Construction, Yangtze University, Jingzhou 434000, China; 201671366@yangtzeu.edu.cn (J.Z.); 201672327@yangtzeu.edu.cn (Y.L); 2 Department of Mechanical Engineering, University of Houston, Houston, TX 77204, USA

**Keywords:** L-shaped concrete filled steel tube (L-CFST) column, piezoceramic transducers, smart aggregate (SA), active sensing, wavelet packet-based damage index, low-frequency cyclic loading

## Abstract

L-shaped concrete filled steel tube (L-CFST) columns are used frequently in civil engineering, and the concrete damage inside the L-CFST column is difficult to monitor. This research aims to develop a new method to monitor the internal concrete damage in the L-CFST column by using embedded piezoceramic smart aggregates (SAs) under low frequency cyclic loading. The SA enabled active method is used to monitor the concrete damages near the bottom of the L-CFST columns, and the wavelet packet analysis is used to establish a damage index, which is used to analyze the acquired data. During the experiment, three L-CFST columns with different wall thickness of the steel tube were tested. The experimental results find that the structural damage indices under the low-frequency cyclic loading are basically consistent with the results of the hysteretic curves and the skeleton curve of the specimens, and are in good agreement with the experimental phenomena. We conclude that the use of smart aggregate can directly and clearly reflect the damage process of the concrete core, demonstrating the feasibility of using piezoceramic smart aggregates to monitor the internal concrete damage of the L-CFST column.

## 1. Introduction

In recent years, high performance and multi-functional materials and structures have received much attention [1,2,3,4]. Among them, concrete-filled steel tube (CFST) columns, which have characteristics such as high stiffness and strength, large energy absorption capacity, and high ductility, are widely used in the construction of buildings. There are a number of studies on the CFST columns, such as Shams et al. [5], Portolés et al. [6], Gourley et al. [7], Lee et al. [8], Sakino et al. [9], and Varma et al. [10,11,12]. L-shaped concrete-filled steel tube (L-CFST) columns are increasingly used because of their architectural appearance and space saving at structural corners. Wang et al. [13], Shen et al. [14], and Zhang et al. [15] investigated the seismic behavior of L-CFST columns. CFST structures are subject to damages under the repeated action of seismic loads. Internal structural damages will continue to accumulate with the cyclic action of loads and the structure will fail as the damages increase to a certain degree. The damage of concrete inside the steel tube will significantly reduce the bearing capacity and ductility of the CFST, however, the internal concrete damage cannot be directly observed. Therefore, it is very important to monitor the internal concrete damage of the structure. 

At present, the methods of structural damage detection include X-ray, acoustic emission, and ultrasonic methods [16,17], among others. Carpinteri et al. [18] used acoustic emission (AE) technology to evaluate visual cracks in reinforced concrete of a multi-story building. Behnia et al. [19] reviewed the application of acoustic emission technology to the health monitoring of concrete structures. Rucka and Wilde [20] studied the cracks of reinforced concrete structures under tensile stress by the ultrasonic method. Antonaci et al. [21] used linear or nonlinear ultrasonic methods to detect the compressive damage of circular concrete columns. Yue et al. [22] developed a damage detectability model of pitch-catch configuration using lamb waves for composite plates 

In recent years, smart materials, such as optical fibers and piezoelectric materials [23,24,25], have been successfully applied to the health monitoring of structures. Kerrouche et al. [26] used embedded Bragg grating optical fiber sensors to monitor the strain on carbon fiber polymer reinforcement (CFPR) rods. Ho et al. [27] proposed a smart anchor plate, a simple but effective device that uses a fiber Bragg gratings (FBG) sensor to monitor the load level of the rock bolt. DeäŸErliyurt et al. [28] used FBG sensors to monitor the damage of the composite beam structure under bending. Tjin et al. [29] placed FBG strain sensors in a reinforced concrete structure to monitor strain changes in loading and unloading tests at different locations within the structure. Piezoceramic transducers, including the piezoceramic smart aggregates, have features such as low cost and actuating-sensing functions, and are widely used in structural health monitoring research [30,31,32,33,34]. Sharif-Khodaei at al. [35] employed piezoelectric transducers to detect impact damages to a composite plate. Song et al. [36] embedded piezoelectric smart aggregates into reinforced concrete beams to perform damage monitoring through destructive tests. Du et al. [37] embedded the piezoceramic smart aggregates (SAs) into a quartz sand-filled steel tube column (SFSTC) to monitor the internal structural stress during impacts. Li et al. [38] studied the damage of concrete beams under a three-point bending test, and compared the damages measured by acoustic emission sensors with the damages measured by smart aggregates. Du et al. [39] used piezoceramic transducers to perform the damage detection of pipeline with multi-cracks. Feng et al. [40] used embedded smart aggregates to monitor the internal damage of concrete piles, including cracks, partial mud intrusion, secondary pouring, and all mud intrusion to four kinds of damage. Kong et al. [41] used SAs to study the early hydration characteristics of concrete, and then divided the early hydration of concrete into three states: fluid state, transitional state, and hardening state. Markovic et al. [42] established a model of damage detection process for concrete beams based on piezoelectric smart aggregates. Du et al. [43] investigated the pipeline corrosion pit with the time reversal method using piezoceramic transducers. Chalioris et al. [44] used embedded and externally bonded piezoelectric transducers to evaluate a shear-critical reinforced concrete beam. Nestorović et al. [45] proposed a numerical modeling of the damage detection process in a concrete beam with piezoceramic transducers. Olmi et al. [46] studied the use of embedded piezoelectric sensors to monitor impact when an over-height truck collides with a reinforced concrete beam. Ghafari [47] studies the feasibility of using piezoelectric sensors to characterize the compressive strength of cement paste mixed with additional cementitious materials.

Piezoelectric transducer based structural damage detection methods include two major categories: the electromechanical impedance method [48,49,50] and the active sensing method. Yang et al. [51] used the structural mechanical impedance (SMI) extracted from the PZT (Lead Zirconate Titanate) electro-mechanical (PZT EM) admittance signature as the damage indicator. Providakis and Voutetaki [52] investigated a statistical metamodeling utilization of electro-mechanical admittance approach to the damage identification. Xu et al. [53] applied the impedance method to monitor the damage of a structure by using both embedded and surface bonded piezoelectric transducers. Madhav et al. [54] reviewed and prospected the application of electromechanical impedance technique in engineering structures. Karayannis et al. [55] proposed a method for monitoring the potential damage of reinforced concrete members by electromechanical admittance method, which used a bonded piezoelectric sensor. Wang et al. [56] proposed a new detection method based on an inverse impedance method to study the damage detection of plain concrete beams. Interestingly, Zou and Aliabadi (2015) developed a piezoelectric sensor for damage detection with self-diagnosis capacity using electro-mechanical impedance.

The active sensing method using piezoceramic smart aggregate is commonly used for monitoring concrete structural damage. The active sensing method uses a smart aggregate as an actuator and another smart aggregate as a sensor. Using the inverse piezoelectric effect, the smart aggregate actuator generates a stress wave, which propagates along the interior of the structure to the piezoelectric smart aggregate sensor. Using the direct piezoelectric effect, the sensor converts the received stress wave into an electrical signal effect. Once there are cracks inside the structure, the signal received by the piezoelectric smart aggregate sensor will attenuate. Therefore, the internal damage of the structure can be analyzed. Gu et al. [57] used SA enabled active sensing monitor concrete early age strength development. Divsholi and Yang [58] used a combination of embedded and surface bonded piezoceramic transducers to detect the damages of a concrete beam, and the results show that this combination provides an effective way to assess both the local and overall damage conditions of the structure. Zou et al. [59] used SA enabled active sensing to monitor the degree of water seepage of concrete structures. Feng et al. [60] proposed an active sensing method based on SAs to monitor cracks and further leakage of concrete pipes. Kong et al. [61] studied the presence of internal moisture in concrete structures and used active sensing methods based on embedded piezoelectric sensors to monitor the presence of cracks and moisture in concrete structures. Xu et al. [62] used a smart aggregate embedded in the concrete and PZT patches bonded on the surface of the steel tube to monitor the debonding between the steel tube and the concrete. Jiang et al. [63] presented a stress wave based active sensing approach using piezoceramic transducers to monitor grouting compactness in real time.

From the above applications, it can be found that the existing smart aggregate-based damage studies are mostly conducted under static loading, however, less experimental studies are carried out under dynamic loading. Gu et al. [64] embedded smart aggregates inside circular reinforced concrete columns to study the internal damages of structures under seismic actions. The research results show that smart aggregates have great potential for application in the health monitoring of mass concrete structures. Liao et al. [65] used smart aggregates to monitor the damage of reinforced concrete frame structures under earthquake excitations and compared the index of smart aggregate monitoring damage with the calculated displacement ductility demand of structural components. The results show that the two are consistent. Kong et al. [66] used smart aggregate to monitor the internal damage of reinforced concrete bridge columns under pseudo-dynamic loading. The results verified the effectiveness of smart aggregates in the health monitoring of reinforced concrete column. Liao et al. [67] conducted tests of concrete columns using smart aggregates for structural health monitoring.

At present, the study of structural damage detection under dynamic loads using SAs is mainly about reinforced concrete structures, and there is little research on the internal concrete damages of concrete filled steel tube structures. CFST structure is a structure filled with concrete inside the steel tube, and its bearing capacity is shared by the steel tube and the internal concrete. The damage of the concrete core will greatly impact the bearing capacity of a CFST. In this research, three L-CFST columns with different wall thicknesses of steel tube were used in this experiment. Piezoelectric smart aggregates were embedded inside the L-CFST columns. The active sensing method based on SA was used to monitor the concrete damage inside column base of L-CFST columns under low-frequency cyclic loading, and wavelet packet analysis was used to establish a damage index, which was then used to analyze and process the monitoring data. The experimental results find that the structural damage index under the low-frequency cyclic loading is basically consistent with the results of the hysteretic curves and the skeleton curve of the specimen. The use of smart aggregate enabled active sensing can directly and clearly reflect the damage process of the concrete core in the L-CFST specimens.

## 2. Experimental Setup

### 2.1. Design of Specimens

**Figure 1:**
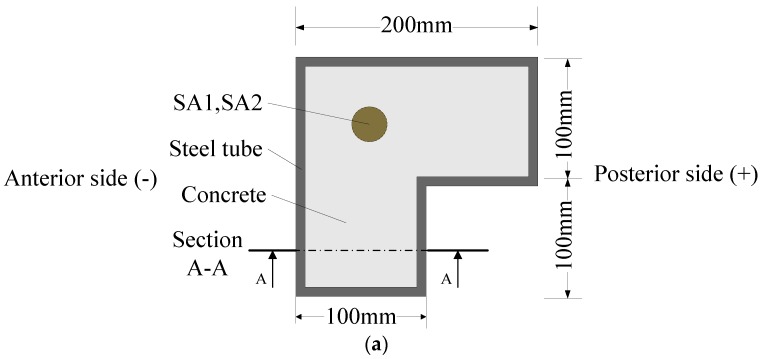

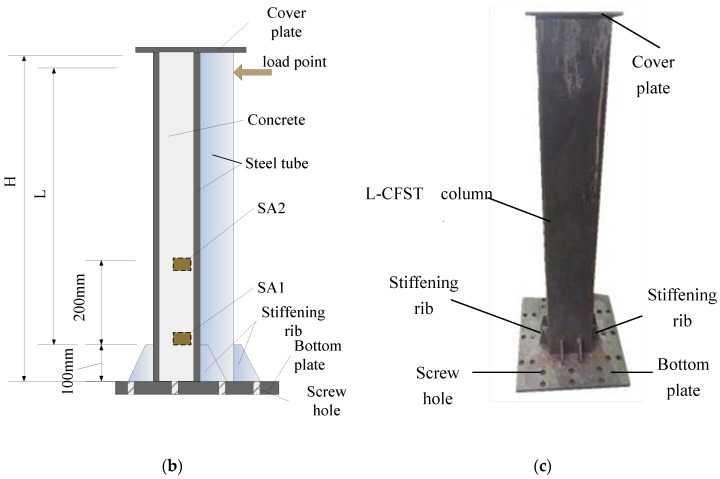
The L-shaped concrete filled steel tube (L-CFST) column specimen. SA—smart aggregates. (**a**) Cross-sectional drawing of the specimen; (**b**) A-A Sectional drawing; (**c**) A photo of the specimen.

Three L-CFST columns were designed and fabricated in the research with the consideration of the cost. The limb length of all of the columns is 200 mm with the length of 1300 mm and the width of 100 mm. The wall thicknesses of the columns are 3 mm, 4 mm, and 5 mm, respectively. The shop drawings of the specimen are shown in Figure 1a–c. The specific parameters of the prepared specimen are shown in Table 1, where *λ* is the slenderness ratio of the specimen, which is defined as

*λ* = *L*/*i*

where *L* is the effective length of the test specimen and *i* is the radius of revolution of the test specimen, and is defined as

$$i=\sqrt{I/A}$$

where *I* is the moment of inertia of the specimen and *A* is the cross-sectional area of the specimen. Please note that the Q235 steel is used to fabricate all the specimens.

**Figure 2:**
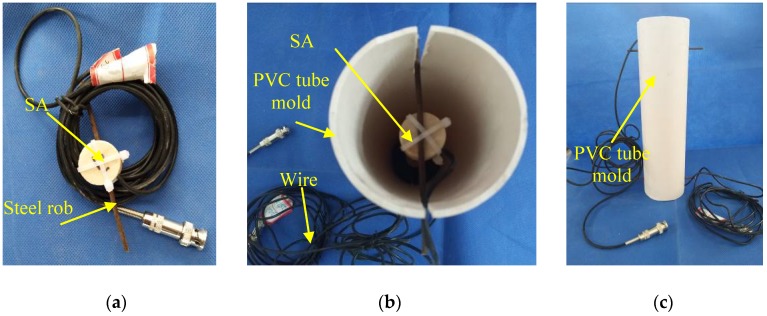
The procedure of embedment of SA. (**a**) The SA prior to embedment; (**b**) The placement of SA; (**c**) A photo of the PVC mold.

As the first step to fabricate the L-CFST specimens, two smart aggregates (SAs) were tied to steel bars, as shown in Figure 2a, and then fixed at the center of a 75 mm diameter PVC (Polyvinyl Chloride) tube mold, into which the concrete was poured. The distance between the two smart aggregates is 200 mm, as shown in Figure 2b,c. After the concrete was poured and solidified, the mold was removed and placed at the bottom of L-CFST column, as shown in Figure 1. Then, as the second step to fabricate the specimens, the concrete was poured in the mold of the L-CFST column. The concrete strength level of the poured concrete is the same as that of the concrete in the previous mold. The curing of the specimen and the welding of the cover plate after the pouring were completed. The geometric center of the cover was aligned with the geometric center of the hollow steel tube.

### 2.2. Properties of Materials

The steel plates used in the tests were processed into standard test specimens, each with different thicknesses of 3 mm, 4 mm, and 5 mm, for tensile testing. The test method is carried out according to the relevant provisions of the Chinese standard (GB/T228-2002), and the yield strength *f_y_* and the ultimate strength *f_u_* are shown in Table 2.

The C45 commercial concrete was poured into the tube, and three standard concrete cubes with the dimensions of 150 mm × 150 mm × 150 mm were prepared at the same time. The test blocks and L-CFST column were naturally cured under the same conditions. The compressive strength of the concrete cube in the steel tube is measured by a cube test block. The mean value of the compressive strength of the concrete standard test block measured was *f_cu_* = 48.35 Mpa on the 28th day, and the mechanical properties of the concrete are shown in Table 3.

### 2.3. Methods of Test Loading and Data Acquisition

**Figure 3:**
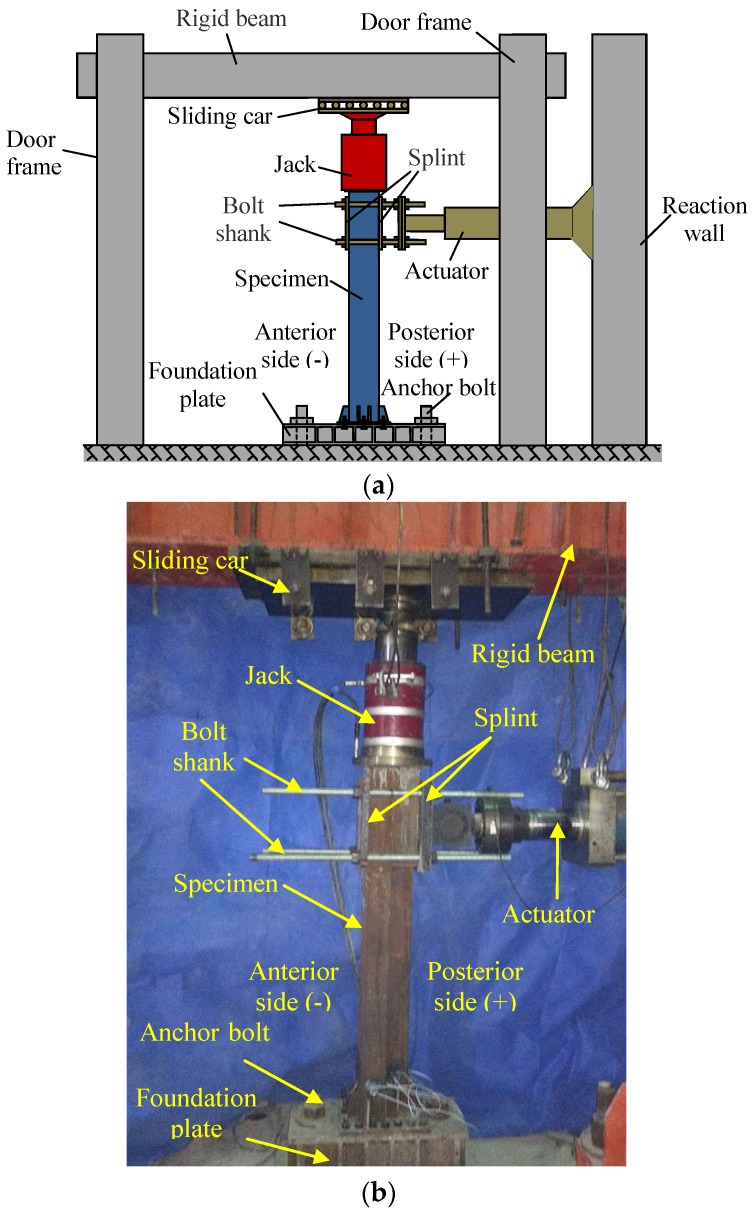
The loading setup and the specimen. (**a**) Schematic of testing apparatus and a specimen; (**b**) A photo of the testing setup and a specimen.

The schematic and physical diagram of this test device are shown in Figure 3a,b. The maximum displacement of the actuator is 500 mm. The axial load is applied to the top of the specimen through a 1000 kN jack.

**Figure 4:**
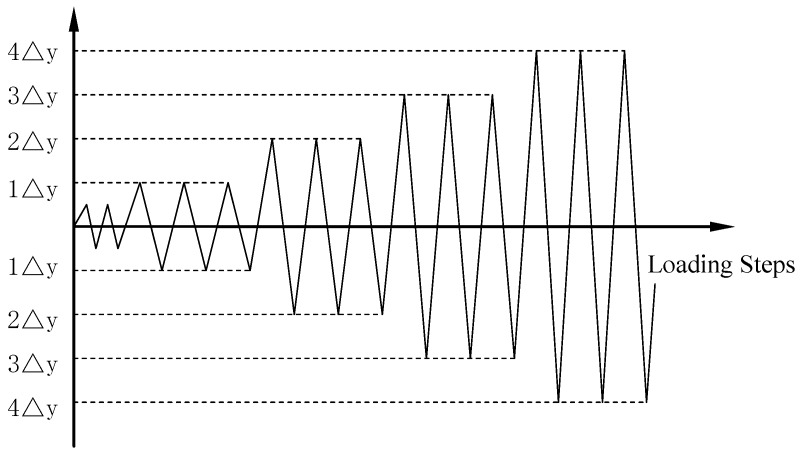
The test loading schedule.

Before the test, preloading along the axial direction was applied twice to stabilize the load and deformation of the specimen, and then the vertical axial force is increased to the full load. Preloading was also applied laterally twice to eliminate the non-uniformity in the steel column and to check the performance of the instrumentation. The displacement control loading method was used in this test. First, the yield displacement of the specimen was estimated. At the initial stage of loading, when the displacement did not exceed the yield displacement, the loading displacement was loaded in incremental increments of less than 5 mm, and each load was performed once. When the displacement exceeded the yield displacement, the control load was applied with a multiple of the displacement, and each loading displacement was repeated three times until the lateral loading force of the specimen fell below 85% of the peak loading force, and then the loading was stopped. The specimen was considered to have been totally damaged at this stage. The test loading schedule is shown in Figure 4.

**Figure 5:**
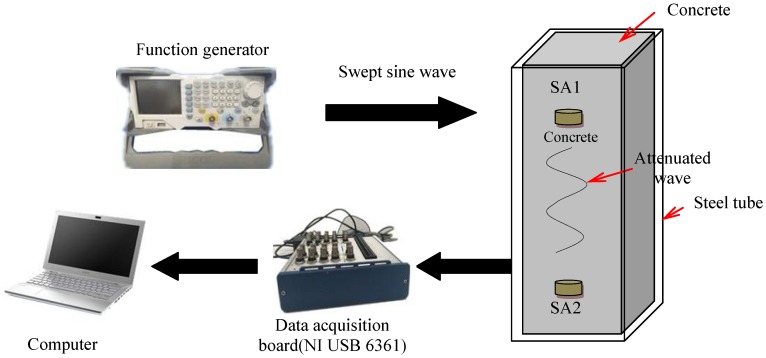
Schematic of experimental instrumentation.

In the three specimens of this research, two SAs were embedded inside each specimen. The SAs are named SA1–SA6 according to the serial number of the components and are shown in Table 4. Upon excitation from a signal from a function generator with amplification, the smart aggregate actuator generates stress waves, and the stress wave is transmitted to the piezoelectric smart aggregate sensor along the interior of the structure. The output signal of the sensor is collected by the data acquisition board (NI USB 6361). The schematic of experiment instrumentation is shown in Figure 5, and the distance between the two embedded SAs is 200 mm. SA1, SA3, and SA5 are used as actuators, and SA2, SA4, and SA6 are used as sensors to monitor the damage of the concrete inside the specimens. Data of the internal damage of the structure can be retrieved by analyzing the electrical signals output by the piezoelectric smart aggregates. The frequency and period are shown in Table 5. In the process of cyclic loading of the specimen, the signal is collected for each displacement peak (the specimen is loaded to the peak of each displacement amplitude) and 0 point (the specimen is loaded to peak where the displacement is 0). The peak displacement is divided into the positive peak displacement and the negative peak displacement, which are labeled in Figure 4.

## 3. Results and Discussion

### 3.1. Experimental Phenomena

**Figure 6:**
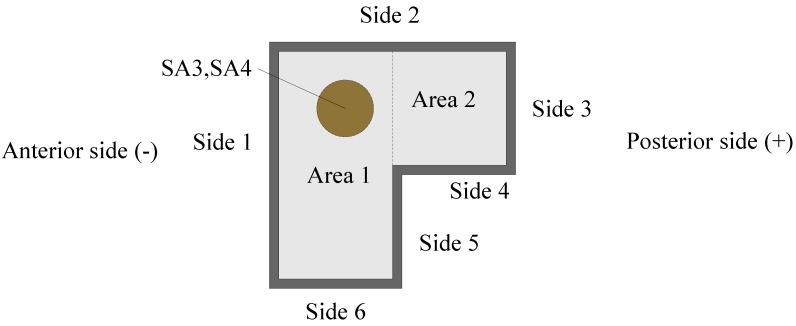
Details of the specimen—a cross-sectional view.

**Figure 7:**
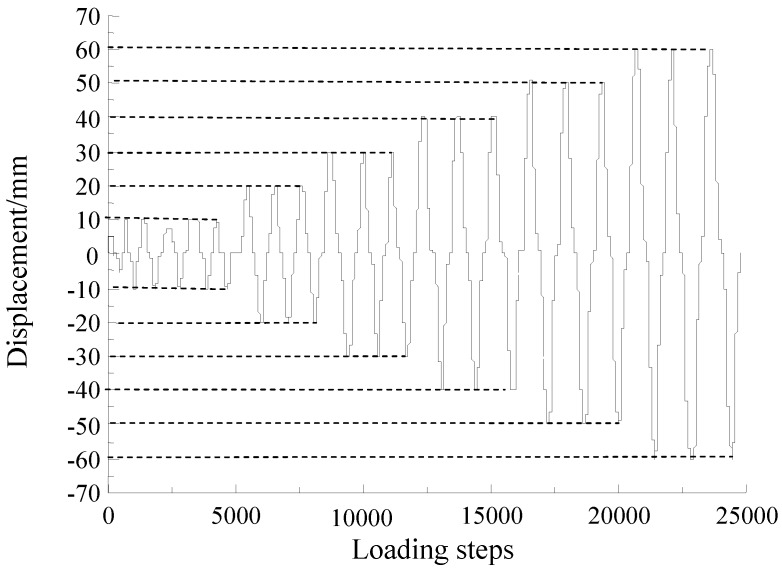
The loading schedule of L-CFST2 (in terms of displacement).

During the loading process, the anterior side of the test specimen is defined as the negative direction and the posterior side is defined as positive. The cross-section of the specimen is numbered to facilitate the description of the test phenomenon of the specimens, as shown in Figure 6. The loading conditions are shown in Table 6. The test loading schedule is shown in Figure 7.

**Figure 8:**
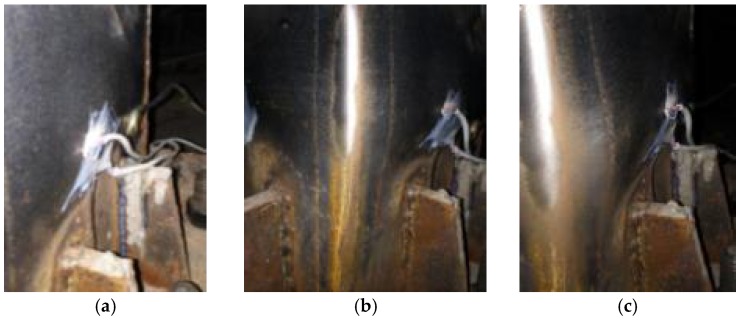

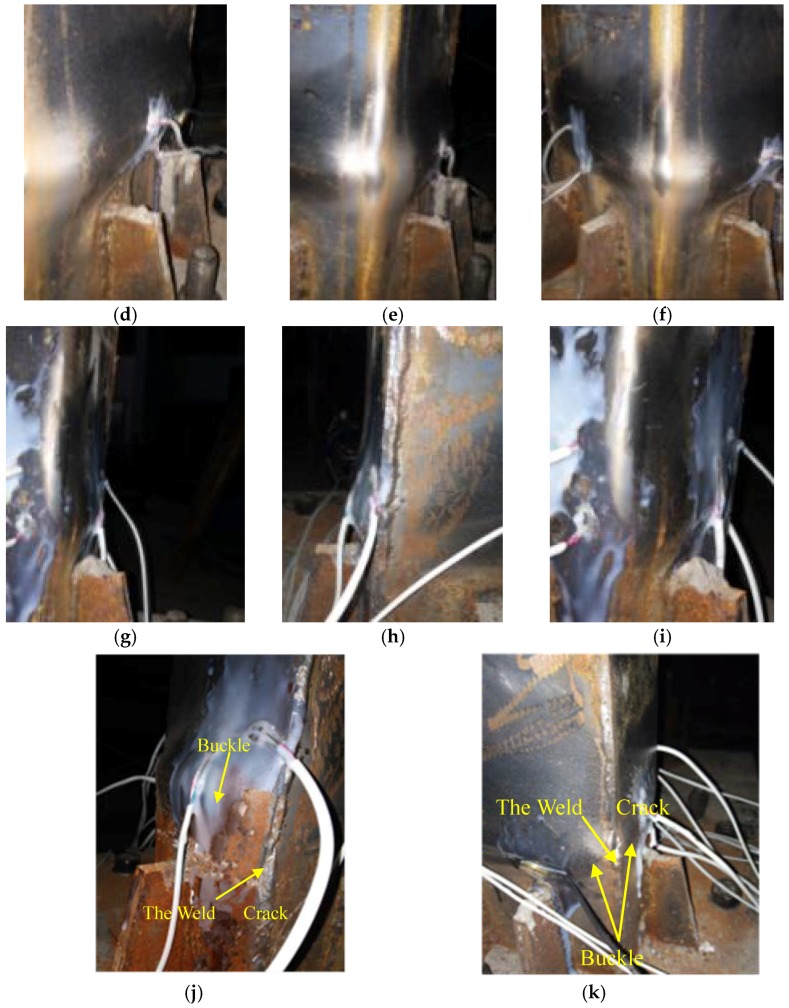
Sectional view of damages of L-CFST2. (**a**) Condition 8 slight buckle in Side 1; (**b**) Condition 12 buckle in side 1; (**c**) Condition 18 severer buckle in side 1; (**d**) Condition 2 severer buckle in side 1; (**e**) Condition 30 severer buckle in side 1; (**f**) Condition 34 severer buckle in side 1; (**g**) Condition 13 severer buckle in side 3; (**h**) Condition 20 severer buckle in side 2; (**i**) Condition 25 severer buckle in side 4; (**j**) Condition 32 severer buckle in side 6, the weld cracked; (**k**) Condition 32 the weld cracked.

The test phenomenon of the specimen L-CFST2 is described as follows, and the test phenomena of other specimens are basically similar. During working conditions 1–6, the specimen has no obvious change, and during working condition 8, side 1 of the specimen appears slightly buckled. During working condition 13, the side 3 of the specimen appears slightly buckled, and during working condition 14, the negative lateral load of the specimen reaches the peak (Pmax = −102.272 kN). At working condition 20, side 2 of the specimen appears slightly buckled, and at working condition 25, side 4 of the test piece specimen appears buckled and the lateral load reaches its peak (Pmax = 110.920 kN). At working condition 32, side 6 of the test specimen appears buckled and the weld of the specimen is cracked. At working condition 35, the weld of the specimen is cracked, and the lateral load of the specimen has dropped to 85% of the peak value, and the experiment stops loading. In the test, the buckle locations of the specimen side are found to be about 5–9 cm above the stiffener, and the buckle of the side increases with the subsequent load. The images of the buckle on the side are shown in Figure 8a–k.

### 3.2. Hysteretic Curves and Skeleton Curves

The lateral force-displacement curve under the low-frequency cyclic loading clearly shows the hysteretic effect. The hysteretic loops effectively reflect the seismic performance of the specimen, including the energy dissipation performance of the specimen. A skeleton curve refers to an envelope curve of hysteresis loops and is drawn by concatenating the peak points of the first cycle. The skeleton curve shows the peak point trajectory of the horizontal force of the CFST column under various displacements, and it reflects the ductility and strength change of the specimen during the entire loading process.

**Figure 9:**
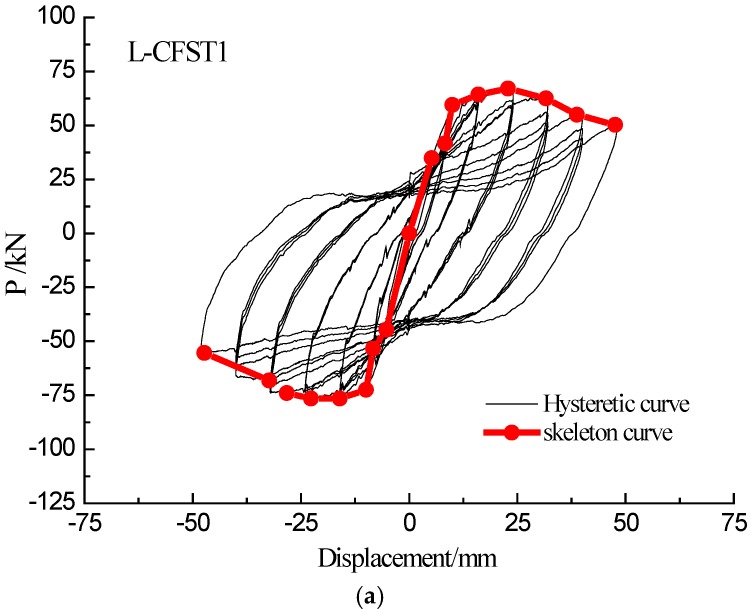

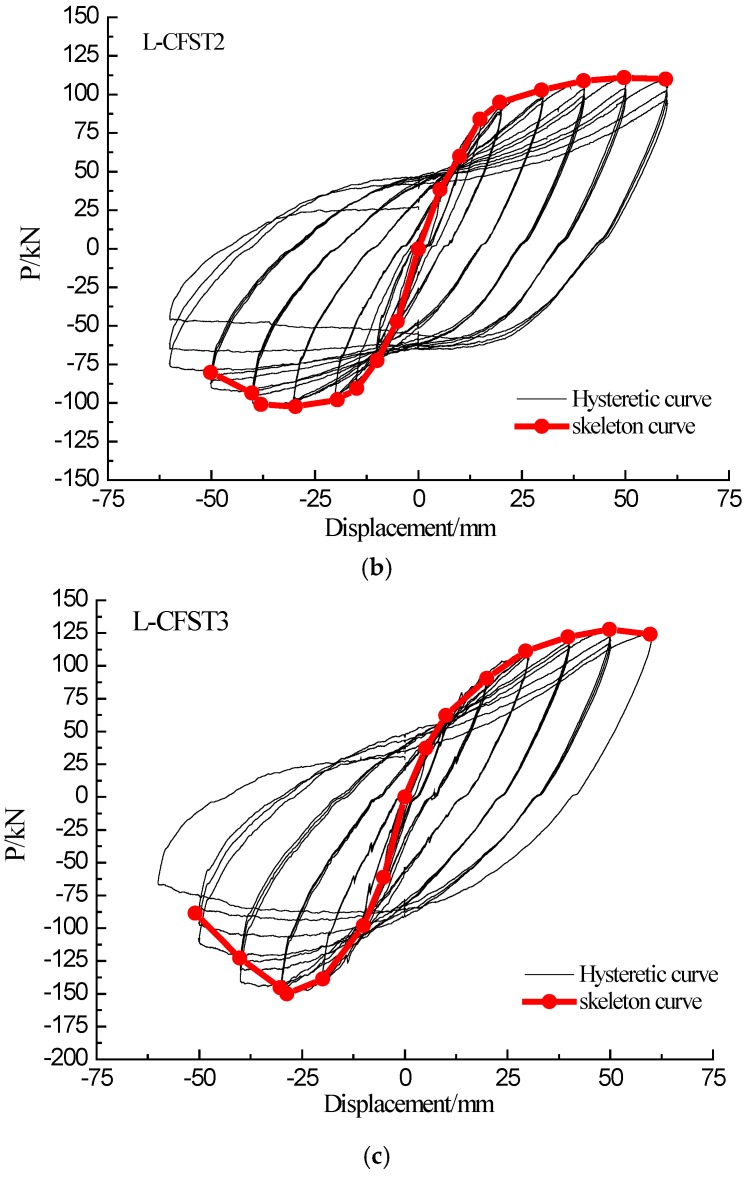
Hysteretic curves and skeleton curves. (**a**) L-CFST1; (**b**) L-CFST2; (**c**) L-CFST3.

The trends of the hysteresis loops and corresponding skeleton curve development of the three test specimens are basically the same in the tests. The hysteresis curves and skeleton curves of all the three specimens are shown in Figure 9a–c, respectively. Analyzing the hysteresis loops and the skeleton curve of the three specimens reveals that the slope of the load-displacement curve changes only slightly at the initial stage of loading, and the loading curve basically coincides with the unloading curve of the specimen. At this stage, the specimen is in the elasticity stage. As the loading continues, the slope of the hysteresis curve gradually increases, and the specimen enters the yielding stage. With the further increase of the load, the lateral load of the specimen reaches the peak value, and then the lateral load gradually decreases as the load continues. In the initial stage of the loading of L-CFST column specimens, the damage to the internal concrete is limited because of the restraint provided by of the outer steel tube of the specimen. With the increase of the loading displacement and the number of cycles, the external steel tube gradually yields and the confinement effect of the steel tube on the internal concrete decreases. As a result, the number of cracks in the concrete increases, the width of the crack increases continuously, and the effective area of the concrete in the compression zone decreases accordingly. Therefore, the bearing capacity of the concrete reduces gradually. At the same time, the bite-engagement between the concrete and the aggregates is gradually deteriorating because of the effect of the reciprocating load, and the cracks repeatedly open and close under the cyclic loading.

The skeleton curve and hysteresis loops of L-CFST2 specimen are described here, and the other specimens are basically the same. The skeleton curve and hysteresis loops of the L-CFST2 specimen are shown in Figure 9b. It shows that the skeleton curve is basically a straight line when the specimen loading displacement is between 0–10 mm, and the L-CFST2 specimen is in the elastic stage from the skeleton curve. At this time, the external steel tube has not been yielded, and the external steel tube has a large constraining effect on the concrete core of specimen column. The cracks and damages of the concrete core increase slowly. It also shows that there is no buckle in the external steel tube during the loading process based on the experimental phenomenon. When the specimen loading displacement is between 10 mm–50 mm, the skeleton curve shows a nonlinear saturating curve. At this time, the specimen is in the yielding stage, the steel tube starts to buckle gradually, and the confinement effect of the steel tube on the internal concrete decreases, causing the gradual increase of the damages in the concrete core area. The lateral load reaches the peak when the displacement is 50 mm, then the load value continues to fall, and the skeleton curve is in the descending section, and the weld cracks. As a result, the internal concrete completely losses its compressive capacity.

**Figure 10:**
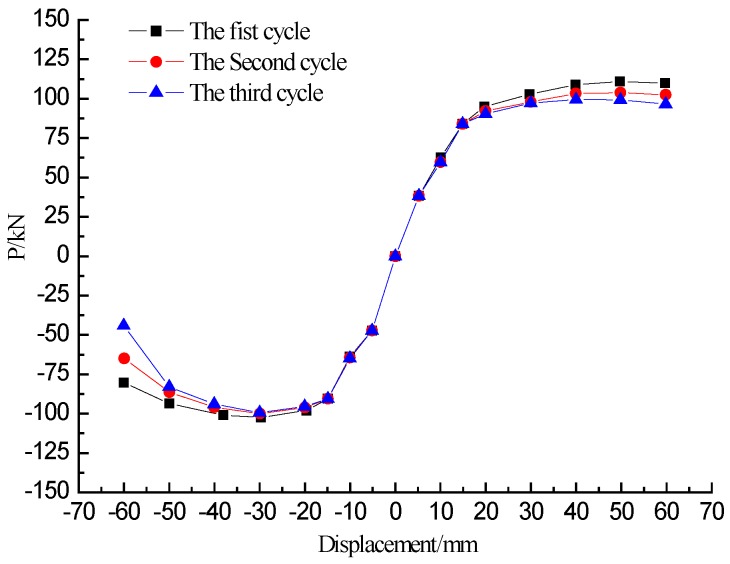
Three cyclic skeleton curves of L-CFST2.

The three cycles of the skeleton curve of each amplitude of the specimen L-CFST2 are plotted in Figure 10, which illustrates that the second cycle and the third cycle skeleton curves are almost coincident with the first one of the specimen at the initial stage of loading, and the stiffness of the specimen does not significantly degrade. When the specimen yields, the stiffness degradation of the specimen worsens with the displacement, however, not very obviously. The reason for this phenomenon lies in the restraining effect of the steel tube on the concrete core, which leads to the increased loading capacity and the improved plastic and toughness properties. The presence of concrete delays or avoids local buckling of the steel tube, enhancing the stability of the L-CFST column.

**Figure 11:**
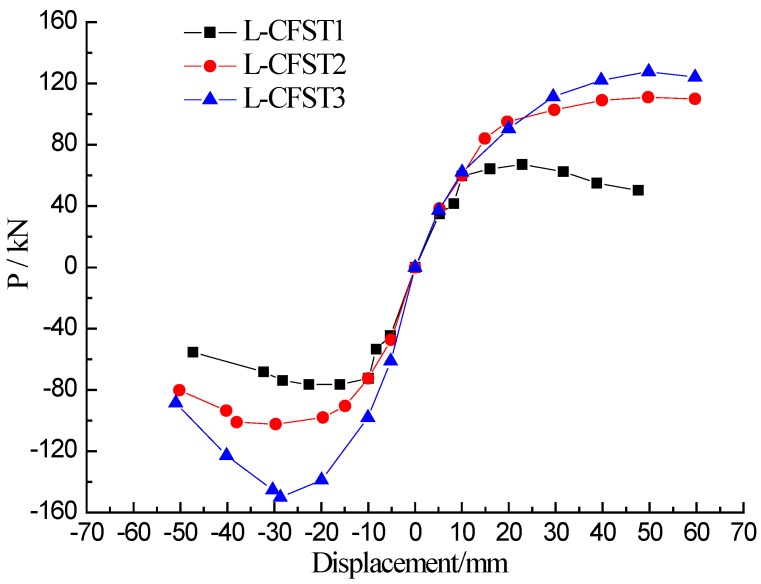
Skeleton curves of three specimens.

The skeleton curves of the three specimens are shown in Figure 11. The trend of the skeleton curves of the three specimens is basically the same. Analyzing the skeleton curves of the three specimens reveals that the slope of the load-displacement curve changes only slightly during the initial stage of loading, and the loading curves basically coincide with the unloading curves of the specimen. At this stage, the specimen is in the elasticity stage. As the loading continues, the slope of the skeleton curve gradually increases, and the specimen enters the yielding stage. The specimen L-CFST1 yielded firstly, subsequently, the specimens L-CFST2 and L-CFST3 yielded. The peak displacement of L-CFST1 was the smallest and this specimen was the first to experience total damages. It shows that the increase of the wall thickness of the steel tube can effectively increase the ultimate bearing capacity of the specimen, and the constraint of the steel tube on the internal concrete will also be effectively improved.

### 3.3. Damage Detection Using Active Sensing

In order to monitor the internal damage degree of the specimen, the signal *S* output by smart aggregate sensor is processed by wavelet packet analysis. The signal *S* is decomposed with n-level wavelet packet into 2*^n^* wavelet packet signal sets $\left\{S_{1},S_{2},\cdots ,S_{2^{n}}\right\}$ [68], $S_{j}$ is the decomposed signal and *j* is the frequency band index (*j* = 1, …, 2*^n^*). $S_{j}$ can be represented by

(1) $$S_{j}=\left[S_{j,1},S_{j,2},S_{j,3},\cdots ,S_{j,m}\right]$$

where *m* represents the number of sampling data. In addition, the energy $E_{j}$ of the decomposed signal $S_{j}$ is defined by

(2) $$E_{j}=S_{j,1}{}^{2}+S_{j,2}{}^{2}+\cdots +S_{j,m}{}^{2}$$

At time *i*, the energy vector of the signal can be expressed as

(3) $$\;E_{i,j}=\left[E_{i,1},E_{i,2},\cdots ,E_{i,2^{n}}\right]$$

In this paper, the root mean square index is used to define the internal concrete damage index (*DI*) of the specimen as

$$DI=\sqrt{\overset{2^{n}}{\underset{j=1}{\sum }}{\left(E_{i,j}-E_{h,j}\right)}^{2}/\overset{2^{n}}{\underset{j=1}{\sum }}{\left(E_{h,j}\right)}^{2}}$$

where $E_{h,j}$ represents the energy vector in the healthy state and $E_{i,j}$ represents the signal energy vector at the *j* band index at time *i*.

**Figure 12:**
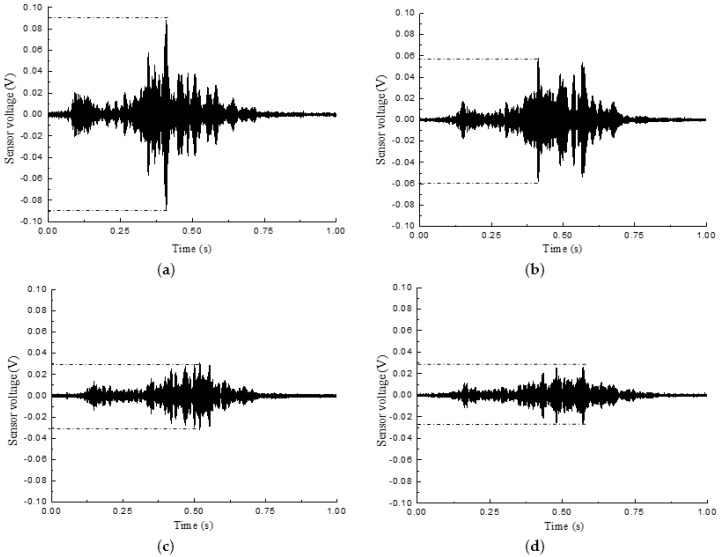

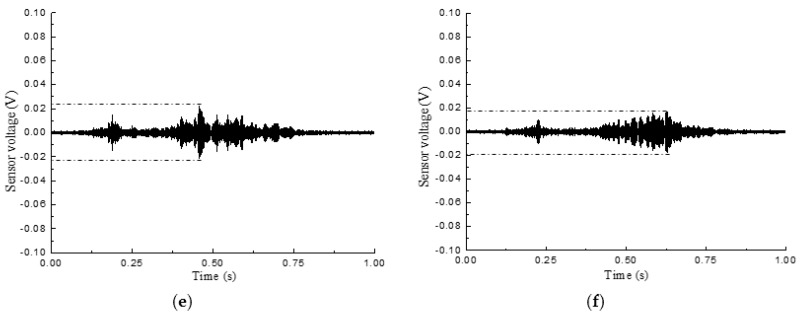
SA4 output voltage signal of L-CFST2. (**a**) SA4 output voltage signal in the healthy state; (**b**) SA4 output voltage signal under 5 mm; (**c**) SA4 output voltage signal under 10 mm; (**d**) SA4 output voltage signal under 15 mm; (**e**) SA4 output voltage signal under 20 mm; (**f**) SA4 output voltage signal under 30 mm.

The output signal of the sensor was processed by wavelet packet analysis, and the DI of each signal was obtained. From Equation (4), we can see that when the energy vector approaches $E_{h,j}$, the signal’s damage index (DI) approaches zero. At this time, it is considered that the specimen has no damage. The greater the damage index is, the more serious the damages are within the structure. When the damage index approaches 1, the structure tends to be completely damaged. In this experiment, each output signal of the sensor was decomposed into 32 wavelet packets using wavelet packet analysis, and the corresponding damage index was obtained. The output signals of the SA4 sensor during the loading of the specimen L-CFST2 are shown in Figure 12a–f, which reveal that the output voltage amplitude of smart aggregates tends to decrease as the loading displacement increases.

**Figure 13:**
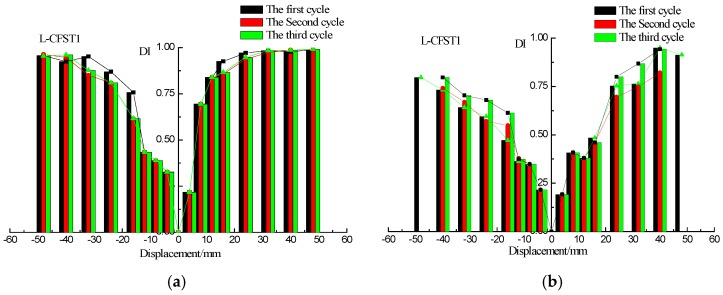
Damage index (DI) vs. displacement for L-CFST1. (**a**) DI vs. positive and negative peak displacements; (**b**) DI vs. zero displacement.

**Figure 14:**
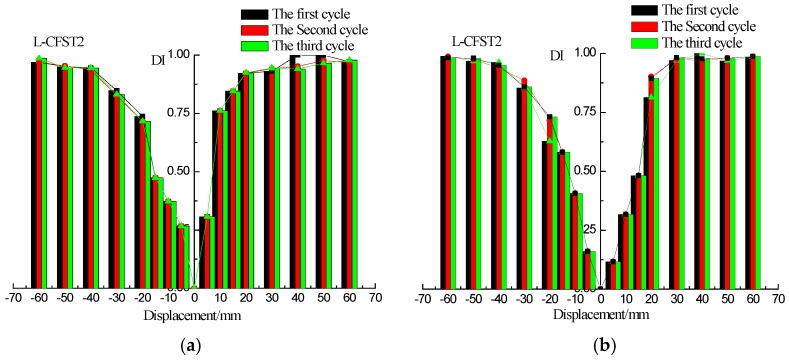
DIs vs. displacement for L-CFST2. (**a**) DI vs. positive and negative peak displacements; (**b**) DI vs. zero displacement.

**Figure 15:**
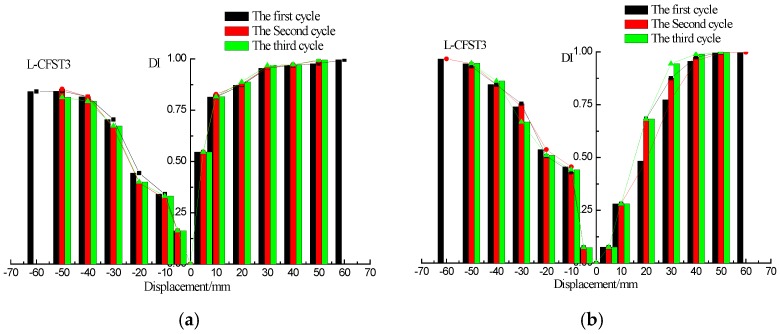
DIs vs. displacement of L-CFST3. (**a**) DI vs. positive and negative peak displacements; (**b**) DI vs. zero displacement.

The wavelet packet-based damage indices of the sensors SA2, SA4, and SA6 are shown in Figure 13, Figure 14 and Figure 15, respectively. The damage index results of L-CFST2 specimen are described here, and those for the other test specimens are basically the same. The DIs with different displacement of L-CFST2 specimen are shown in Figure 14. Figure 14a shows the positive and negative peak displacement-DI graph for each displacement loaded with three cycles during the test. Figure 14b shows the 0 displacement-DI graph for each displacement loaded with three cycles during the test. Figure 14a,b show that the damage index gradually increased with the increase of the loading displacement, and finally saturated towards 1. It suggests that the internal concrete cracks and damages increase with the loading until it is completely damaged. The results of the damage index analysis are basically consistent with those of the previous analyses of the L-CFST2 skeleton curve.

**Figure 16:**
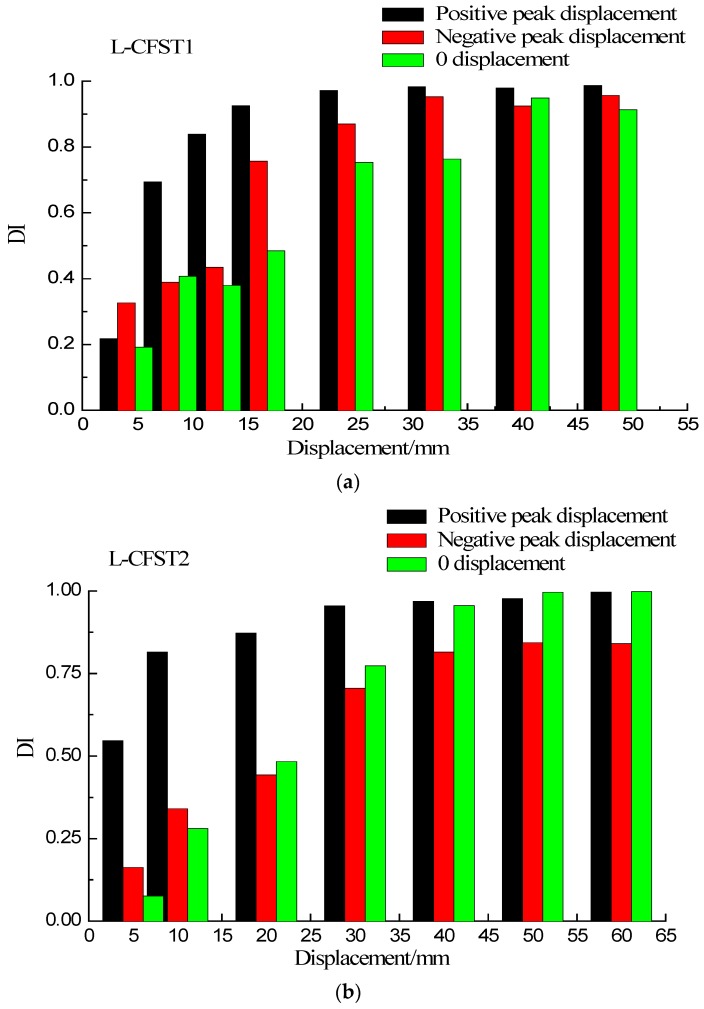

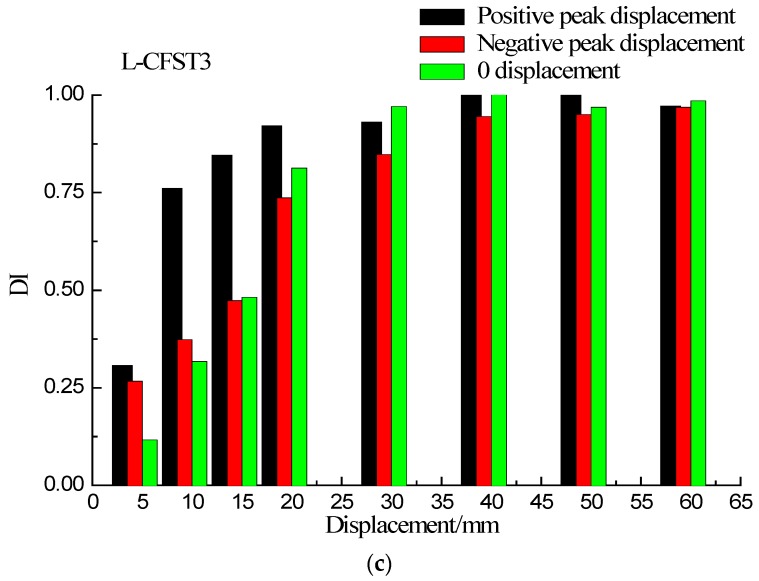
DI vs. displacement of all specimens. (**a**) L-CFST1; (**b**) L-CFST2; (**c**) L-CFST3.

The first cycle damage index graphs of the three test specimens are shown in Figure 16a–c, respectively, for the different displacements (positive peak displacement, negative peak displacement, and zero displacement). Because of the asymmetry of the L-CFST specimen, the concrete in area I (Figure 6) is under tension in the positive peak displacement during the test. Under the negative peak displacement, the concrete in area I is compressed, and the concrete in area II is tensioned. Please notice that the concrete in area II is far from the monitoring position. Therefore, in Figure 16, the value of the damage index at the positive peak displacement is greater than that of the negative peak displacement, and the value of zero displacement damage index is basically between positive and negative peak displacement damage index values, which indicates that the cracking degree of the concrete at the monitoring location is the most serious during the positive peak displacement. This phenomenon reflects the repeated opening and closing of concrete cracks in the core area during the low-frequency cyclic loadings.

Taking the L-CFST2 as an example, the internal concrete damage during the test is analyzed in detail with the help of the DIs. According to Figure 16b, when the positive peak displacement is 10 mm, the damage index value is 0.75, after which the damage index value continues to increase slowly. When the peak displacement increased to 20 mm, the damage value approaches 1. Subsequently, the wavelet packet index tends to be stable and close to 1 as the peak displacement increases. According to the test phenomenon, hysteresis curve and skeleton curve of the L-CFST2 are in the elastic phase in the stage of 0–20 mm, and there is no buckling on the outside surface of the specimen. When the displacement is loaded to 20 mm, a buckle appears in section 1, and the specimen enters the yield phase. The damage index obtained from the SA shows that cracks have already appeared in the concrete of the internal zone 1 when the specimen is in the elastic phase, in which the compressive strength of the concrete under lateral forces is increased because of the constraint of the outer steel tube, and the internal concrete is not completely damaged. When the steel tube enters the yield phase, the confinement effect of the steel tube on the internal concrete is greatly reduced. Then, the value of the damage index basically approaches to 1, and the concrete in the interior area of the specimen is completely damaged.

**Figure 17:**
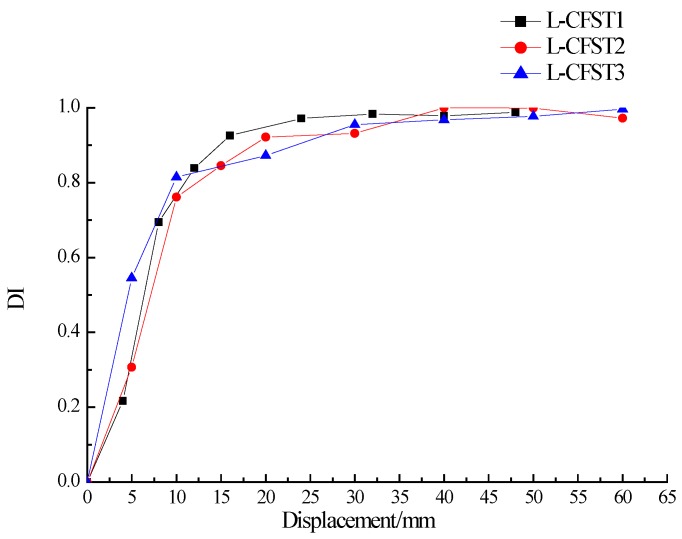
DI vs. positive peak displacement for all specimens.

The positive peak displacement damage index curves of the three test specimens are plotted in Figure 17, which shows that the trend of these damage index curves of the three test specimens are basically the same, and they are all in linear growth at first, then increase slowly, and finally saturate towards 1. According to Figure 10, when the specimen loading displacement is between 0–10 mm, and the three specimens are in the elastic stage. At this stage, the external steel tube does not yield, and the external steel tube has a large constraining effect on the concrete core of specimen column. The cracks and damages of the concrete core increase slowly, and there is no buckle in the external steel tube during the loading process according to the experimental phenomenon. When the specimen loading displacement is between 10–20 mm, the skeleton curve shows a curve line, and the specimen is in the yielding stage. During this stage, the severity of the steel tube buckle gradually increases, reducing the confinement effect of the steel tube on the internal concrete and causing a gradual increase of the concrete damage in the concrete core area. The lateral load reaches the peak when the displacement is 50 mm, then the load value continues to fall with the skeleton curve in the descending section. Eventually, the weld starts to crack. As a result, the internal concrete completely loses its compressive capacity. According to Figure 17, when the specimen loading displacement is between 0 mm–10 mm, the damage index value is between 0–0.75, and then the damage index value continues to increase slowly. When the peak displacement is loaded to 20 mm, the damage value approaches 1. Subsequently, the wavelet packet index saturates towards 1 as the peak displacement increases. After the yielding of the steel tube, the wall thickness of steel tube of L-CFST1 is relatively low, and the restraining effect of steel tube on the internal concrete is the weakest. These reasons lead to the first complete damage of the concrete inside the specimen L-CFST1. The damage index of the specimen L-CFST1 starts to saturate towards 1 at first. The analysis results of the damage index of the specimen are basically consistent with the those of the skeleton curve analysis.

The high cost of fabricating the specimens prevented us from making more specimens. Though this research involves only three specimens of L-shaped Concrete Filled Steel Tubes (L-CFSTs) with different wall thickness, the results of the three specimens are very consistent, which demonstrates that three specimens are enough to demonstrate the proposed method to monitor the internal concrete damages using embedded smart aggregates.

## 4. Conclusions

This research develops a new method to monitor the internal concrete damages in the L-shaped concrete filled steel tube (L-CFST) columns by using embedded piezoceramic smart aggregates (SAs). The SA enabled active method is used to monitor the concrete damages near the bottom of L-CFST columns, and the wavelet packet analysis is used to establish a damage index. Three L-CFST columns with different wall thickness of the steel tube were designed, fabricated, and tested. The analyses of the test results of three L-CFST columns show that the damage index value of the specimen initially increases linearly with the increase of the loading displacement, and then gradually approaches 1, and then it will remain basically unchanged. The displacement-damage index histogram shows that the damage of the concrete inside the specimen increases with the increase of the loading displacement. With the increase of the number of cycles, all three specimens show no significant changes in the concrete internal damage of the specimen under the same displacement amplitude loading. Comparing the positive-negative peak displacement and the 0-displacement index histogram of the damage index, the value of the damage index continuously changes with the loading of the low-frequency cyclic load, which is well reflected by the phenomenon that repeated opening and closing of concrete cracks in the core area in the low-frequency cyclic loading process. The analysis result of the data monitored by the smart aggregate under the low-frequency cyclic load is basically consistent with the analysis results of the hysteresis curve and the skeleton curve of the specimen. The test results demonstrate that the use of smart aggregate can directly and clearly monitor in real-time the damage process of the concrete core, which is not directly visible. It can be concluded that it is feasible to use smart aggregate enabled active sensing to monitor the internal concrete core damage of a concrete filled steel tube structure.

## Figures and Tables

**Table 1:** The specific parameters of specimen. L-CFST—L-shaped concrete filled steel tube.

Serial Number	Wall Thickness/mm	Length *H*/mm	Effective Length *L*/mm	Slenderness Ratio *λ*	Axial force/kN
L-CFST1	3	1300	980	22.22	600
L-CFST2	4	1300	980	22.22	600
L-CFST3	5	1300	980	22.22	600

**Table 2:** Steel parameters. *f_y_*—yield strength; *f_u_*—ultimate strength.

Thickness/mm	Yield Strength *f_y_*/MPa	Ultimate Strength *f_u_*/MPa
3 mm	352.67	464.67
4 mm	360.67	464.00
5 mm	360.00	423.67

**Table 3:** The mechanical properties of the concrete.

Specification Strength	*f_cu_*/Mpa	*f_c_*/Mpa
C45	48.35	36.75

**Table 4:** Number of smart aggregates (SAs).

Specimen	L-CFST1	L-CFST2	L-CFST3
Actuator (internal)	SA1	SA3	SA5
Sensor (internal)	SA2	SA4	SA6

**Table 5:** Parameters of the swept sine wave.

Start Frequency	Stop Frequency	Period	Amplitude
1 kHz	300 kHz	1 s	10 V

**Table 6:** The loading conditions.

Condition #	1	2	3	4	5	6	7	8	9
Load displacement (cm)	10	−10	10	−10	10	−10	20	−20	20
Condition #	10	11	12	13	14	15	16	17	18
Load displacement (cm)	−20	20	−20	30	−30	30	-30	30	−30
Condition #	19	20	21	22	23	24	25	26	27
Load displacement (cm)	40	−40	40	−40	40	−40	50	−50	50
Condition #	28	29	30	31	32	33	34	35	36
Load displacement (cm)	−50	50	−50	60	−60	60	−60	60	−60
